# A Metal Badge Embedded in the Forehead: A Case Report of Military Uniform Safety

**DOI:** 10.7759/cureus.20168

**Published:** 2021-12-04

**Authors:** Sara S Alnufaili, Nouf M Althobaiti, Sami S Almuhaimeed

**Affiliations:** 1 Neurosurgery, King Saud Medical City, Riyadh, SAU

**Keywords:** penetrating head injury, occupational injury, head wound, head trauma, foreign body

## Abstract

Foreign body (FB) injury to the head is not uncommon in medical practice. Various objects have been reported in penetrating head injuries. Depth and location of penetration determine the expected complications and management approach. Here, we describe a case of FB injury to the head by the metal badge of a uniform hat and discuss the medical implications of such injuries among a large population of workers at risk. A 23-year-old male presented to the emergency room with the metal badge of his uniform hat embedded in the left side of his forehead after a physical altercation at work. Imaging revealed FB penetrating the soft tissue and minimally embedded in the outer table of the left frontal bone. The FB was removed in the emergency room with no complications. The wound was then cleaned and sutured, and the patient was discharged home with oral antibiotics for one week. Penetrating FB to the head can present significant morbidity to military personnel, and thus a safer design of work uniforms is warranted.

## Introduction

Various objects have been reported in penetrating head injuries. The exact location and depth of penetration of the foreign body (FB) determine the severity of the injury, associated complication, and appropriate management strategy [1,2].

Occupational injuries contribute to 10% of all traumatic brain injuries [3]. The International Labour Organization (ILO) mandates that all employers “provide and maintain workplaces, machinery, and equipment, and use work methods, which are as safe and without risk to health as is reasonably practicable” [4]. Some dress uniforms of military personnel around the world have metal badges, and here the focus is on metal badges of uniform hats. The badge is sometimes designed with a sharp end on the inner side, and being placed on the head, puts the workers at potential risk of penetrating head injury during physical contact while at work.

Here, we describe a case of FB injury to the head by a metal badge of a uniform hat and discuss the medical implications of such injuries.

## Case presentation

A 23-year-old male who works as military personnel has presented to the emergency department with a metal FB embedded in the left side of his forehead. He was involved in blunt force physical altercation while at work, and the metal badge of his uniform hat got embedded in his forehead. He complained of local pain and denied a history of vomiting, loss of consciousness, or seizure. Examination revealed a fully conscious, hemodynamically stable patient. There was no active bleeding from the wound and no cerebrospinal fluid leak from the nose. Cranial nerve function was intact. The patient did not have any other bodily injuries.

**Figure 1:**
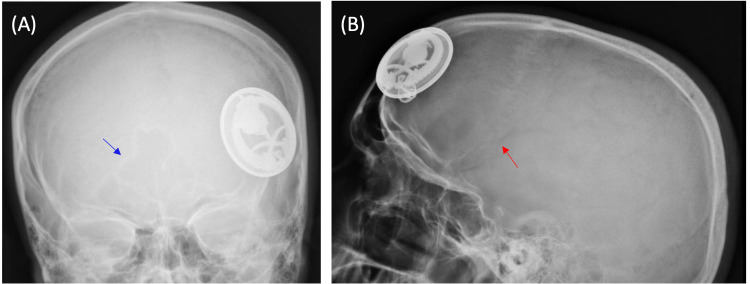
X-ray of the skull coronal view (A) and sagittal view (B) showing a metal foreign body on the left frontal bone. The blue arrow points to the frontal paranasal sinus, while the red arrow indicates the groove of the middle meningeal artery and its branches on the inner side of the temporal-parietal bone.

**Figure 2:**
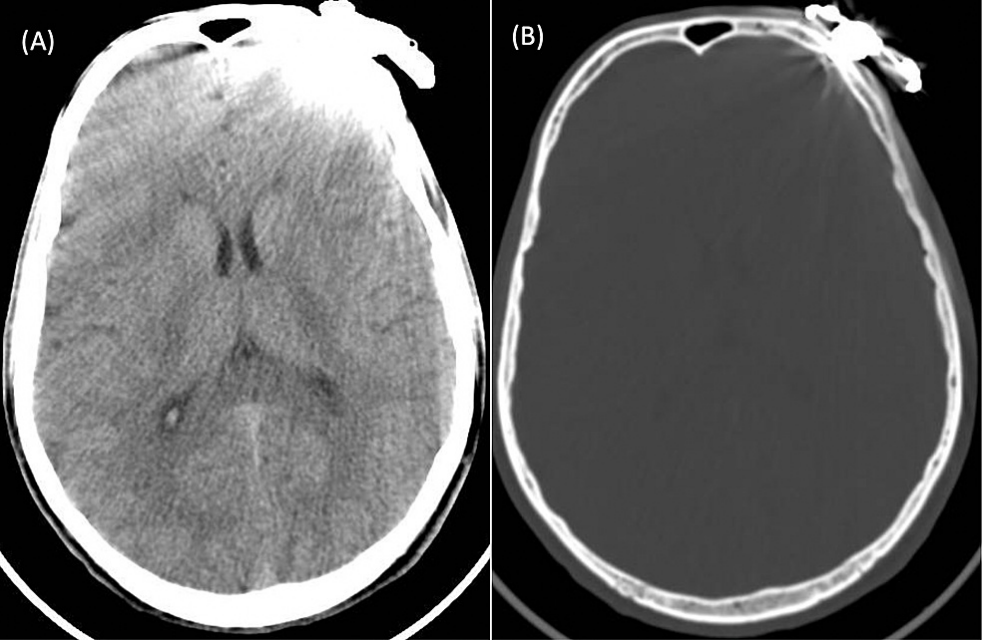
Computed tomography (CT) of the head. (A) Axial view of the brain showing the metal object on the left frontal bone with artifact effect. (B) Axial view of the brain (bone window) showing the metal object minimally embedded in the external table of the frontal bone.

Skull x-ray showed the metal object on the left frontal bone and away from the frontal paranasal sinus (Figures 1A, 1B). Computed tomography (CT) of the head was requested to exclude intracranial penetration. It showed that the FB has penetrated the soft tissue and was minimally embedded on the external table of the left frontal bone (Figures 2A, 2B). No fracture or intracranial hematomas were appreciated.

**Figure 3:**
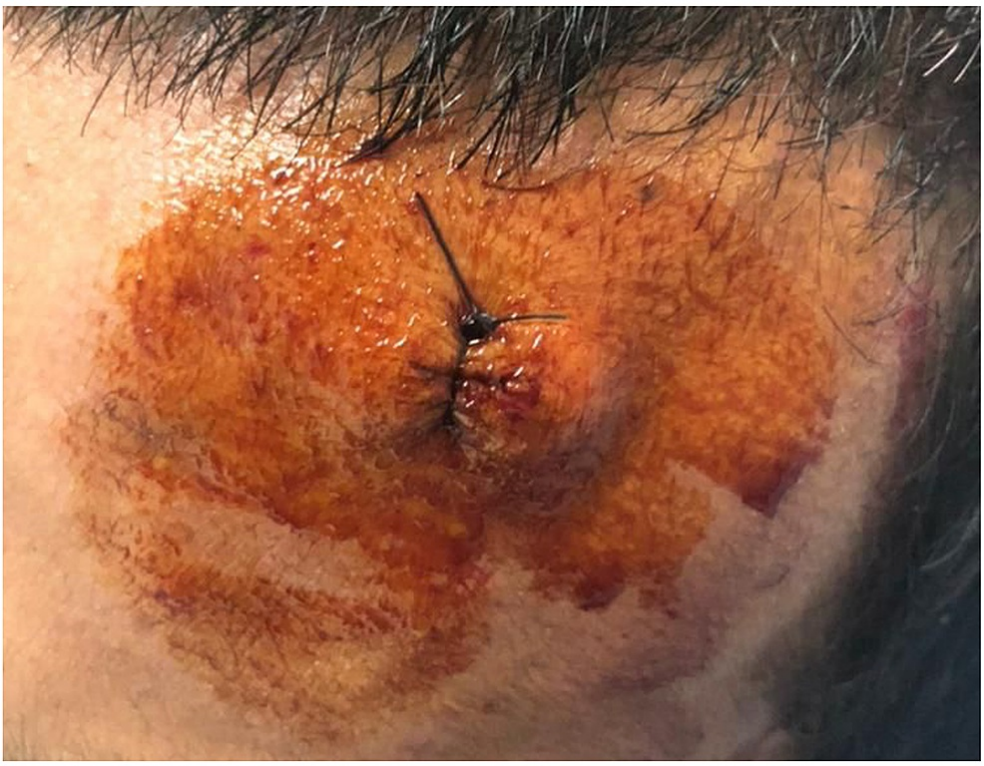
The metal object was removed, and the wound was cleaned and sutured.

The metal object was removed in the emergency room under local anesthesia using a sterile technique with no complications, and the wound was cleaned with normal saline then closed with non-absorbable sutures (Figure 3). The patient was discharged home with oral cephalexin 250mg every six hours for seven days duration.

## Discussion

Different objects such as sharp rods, wooden material, and metal weapons have been reported previously in penetrating head injury [1,2,5]. To our knowledge, this is the first case report of FB injury to the head by a uniform metal badge. Fortunately, our patient had only minimal penetration of the left frontal bone with no intracranial complications. However, the place of the metal badge on the frontal-temporal part of the head bears several risks in case of penetrating injury.

The majority of patients with penetrating head injuries are males [1,2,5]. They present with retained FB and peri-orbital hematoma, CSF rhinorrhea, or other symptoms depending on the site of injury [1,2]. The removal of FB before an appropriate medical evaluation is not advised [1]. Plain skull radiographs are useful in assessing the shape of metal objects and any associated fragments [2] while CT imaging provides more information about FB location, the extent of intracranial penetration, and associated complications [6]. The use of magnetic resonance imaging (MRI) is relatively contraindicated in the case of penetrating metal material [6].

FBs that penetrate cranial bones into brain parenchyma can affect different areas although frontal, temporal, and occipital injuries have better outcomes than other injuries (e.g., injury to the brainstem) [2]. They can result in various complications such as post-traumatic seizure, central nervous system (CNS) infection, traumatic vascular injury, cranial nerve palsies, and vision loss [1,5]. As was mentioned, the location of the metal badge on the head can result in certain complications with penetrating injury. Of these, frontal paranasal sinus fracture with CSF rhinorrhea if the inner part of the sinus is involved. Dural tear and CSF leak increase the risk of CNS infection and the use of peri-operative prophylactic antibiotics minimize the risk of later infection [2]. In addition, the squamous part of the temporal bone is very thin and easily fractured. The middle meningeal artery (MMA) and its branches are running in a groove on the inner side of the temporal bone and can be torn with bone fracture resulting in an epidural hematoma.

The design of military dress uniforms is expected to entail the assessment of potential work hazards and manufacturing of the clothes using the best design and material to prevent these hazards. Working as military personnel involves physical contact with others during training or work duties. Therefore, it is encouraged to have a safer design of uniform metal badges to avoid the above-mentioned injuries.

## Conclusions

This report highlights an under-recognized risk of a common uniform accessory. Penetrating FB to the head can result in bone fracture, infection, and potential vascular injury. Occupational injuries can present minor to major socio-economic implications on the employees and their institutions. The work nature of military personnel warrants a safer design of the metal badge of uniform hats.
